# Diet‐wide association study of 92 foods and nutrients and lung cancer risk in the European Prospective Investigation into Cancer and Nutrition study and the Netherlands Cohort Study

**DOI:** 10.1002/ijc.34211

**Published:** 2022-08-04

**Authors:** Alicia K. Heath, David C. Muller, Piet A. van den Brandt, Elena Critselis, Marc Gunter, Paolo Vineis, Elisabete Weiderpass, Heiner Boeing, Pietro Ferrari, Melissa A. Merritt, Agnetha L. Rostgaard‐Hansen, Anne Tjønneland, Kim Overvad, Verena Katzke, Bernard Srour, Giovanna Masala, Carlotta Sacerdote, Fulvio Ricceri, Fabrizio Pasanisi, Bas Bueno‐de‐Mesquita, George S. Downward, Guri Skeie, Torkjel M. Sandanger, Marta Crous‐Bou, Miguel Rodríguez‐Barranco, Pilar Amiano, José María Huerta, Eva Ardanaz, Isabel Drake, Mikael Johansson, Ingegerd Johansson, Tim Key, Nikos Papadimitriou, Elio Riboli, Ioanna Tzoulaki, Konstantinos K. Tsilidis

**Affiliations:** ^1^ Department of Epidemiology and Biostatistics, School of Public Health Imperial College London London UK; ^2^ Department of Epidemiology Maastricht University Medical Centre Maastricht The Netherlands; ^3^ Biomedical Research Foundation of the Academy of Athens Athens Greece; ^4^ Department of Nutrition and Dietetics Harokopio University Athens Greece; ^5^ Department of Primary Care and Population Health University of Nicosia Medical School Nicosia Cyprus; ^6^ International Agency for Research on Cancer World Health Organization Lyon France; ^7^ Department of Epidemiology German Institute of Human Nutrition Potsdam‐Rehbrücke Bergholz‐Rehbrücke Germany; ^8^ Cancer Epidemiology Program University of Hawaii Cancer Center Honolulu Hawaii USA; ^9^ Danish Cancer Society Research Center Copenhagen Denmark; ^10^ Department of Public Health Aarhus University Aarhus Denmark; ^11^ Division of Cancer Epidemiology German Cancer Research Center (DKFZ) Heidelberg Germany; ^12^ Institute of Cancer Research Prevention and Clinical Network (ISPRO) Florence Italy; ^13^ Unit of Cancer Epidemiology Città della Salute e della Scienza University‐Hospital Turin Italy; ^14^ Department of Clinical and Biological Sciences University of Turin Turin Italy; ^15^ Unit of Epidemiology Regional Health Service ASL TO3 Grugliasco Italy; ^16^ Dipartimento di Medicina Clinica e Chirurgia Federico II University Naples Italy; ^17^ Centre for Nutrition, Prevention and Health Services National Institute for Public Health and the Environment (RIVM) Bilthoven The Netherlands; ^18^ Institute for Risk Assessment Sciences, Division of Environmental Epidemiology Utrecht University Utrecht The Netherlands; ^19^ Julius Center for Health Sciences and Primary Care University Medical Center Utrecht Utrecht The Netherlands; ^20^ Department of Community Medicine, Faculty of Health Sciences UiT‐The Arctic University of Norway Tromsø Norway; ^21^ Unit of Nutrition and Cancer, Cancer Epidemiology Research Program Catalan Institute of Oncology (ICO), Bellvitge Biomedical Research Institute (IDIBELL), L'Hospitalet de Llobregat Barcelona Spain; ^22^ Department of Epidemiology Harvard T.H. Chan School of Public Health Boston Massachusetts USA; ^23^ Escuela Andaluza de Salud Pública (EASP) Granada Spain; ^24^ Instituto de Investigación Biosanitaria ibs.Granada Granada Spain; ^25^ Centro de Investigación Biomédica en Red de Epidemiología y Salud Pública (CIBERESP) Madrid Spain; ^26^ Ministry of Health of the Basque Government Sub‐Directorate for Public Health and Addictions of Gipuzkoa San Sebastián Spain; ^27^ Biodonostia Health Research Institute Epidemiology and Public Health Area San Sebastián Spain; ^28^ CIBER Epidemiology and Public Health (CIBERESP) Instituto de Salud Carlos III (ISCIII) Madrid Spain; ^29^ Department of Epidemiology Murcia Regional Health Council, IMIB‐Arrixaca Murcia Spain; ^30^ Navarra Public Health Institute Pamplona Spain; ^31^ IdiSNA Navarra Institute for Health Research Pamplona Spain; ^32^ Department of Clinical Sciences in Malmö Lund University Malmö Sweden; ^33^ Department of Radiation Sciences, Oncology Umeå University Umeå Sweden; ^34^ Department of Odontology, Section of Cardiology Umeå University Umeå Sweden; ^35^ Cancer Epidemiology Unit, Nuffield Department of Population Health University of Oxford Oxford UK; ^36^ Nutrition and Metabolism Branch International Agency for Research on Cancer Lyon France; ^37^ Department of Hygiene and Epidemiology University of Ioannina School of Medicine Ioannina Greece

**Keywords:** cohort study, diet, foods, lung cancer, nutrients

## Abstract

It is unclear whether diet, and in particular certain foods or nutrients, are associated with lung cancer risk. We assessed associations of 92 dietary factors with lung cancer risk in 327 790 participants in the European Prospective Investigation into Cancer and Nutrition (EPIC). Cox regression yielded adjusted hazard ratios (HRs) and 95% confidence intervals (CIs) per SD higher intake/day of each food/nutrient. Correction for multiple comparisons was performed using the false discovery rate and identified associations were evaluated in the Netherlands Cohort Study (NLCS). In EPIC, 2420 incident lung cancer cases were identified during a median of 15 years of follow‐up. Higher intakes of fibre (HR per 1 SD higher intake/day = 0.91, 95% CI 0.87‐0.96), fruit (HR = 0.91, 95% CI 0.86‐0.96) and vitamin C (HR = 0.91, 95% CI 0.86‐0.96) were associated with a lower risk of lung cancer, whereas offal (HR = 1.08, 95% CI 1.03‐1.14), retinol (HR = 1.06, 95% CI 1.03‐1.10) and beer/cider (HR = 1.04, 95% CI 1.02‐1.07) intakes were positively associated with lung cancer risk. Associations did not differ by sex and there was less evidence for associations among never smokers. None of the six associations with overall lung cancer risk identified in EPIC were replicated in the NLCS (2861 cases), however in analyses of histological subtypes, inverse associations of fruit and vitamin C with squamous cell carcinoma were replicated in the NLCS. Overall, there is little evidence that intakes of specific foods and nutrients play a major role in primary lung cancer risk, but fruit and vitamin C intakes seem to be inversely associated with squamous cell lung cancer.

## Graphical abstract

What's new?

Lung cancer is the leading cause of cancer death worldwide, and while smoking is the most significant factor affecting lung cancer risk, other environmental and genetic factors may contribute. Here, the authors looked for an association between the foods people eat and their lung cancer risk. Using an approach they call a diet‐wide association study, modelled after genome‐wide association studies, they evaluated 92 individual food and nutrient intakes for association with lung cancer risk. No major associations were detected for lung cancer overall, but higher fruit and vitamin C intakes were associated with lower squamous cell lung cancer risk.

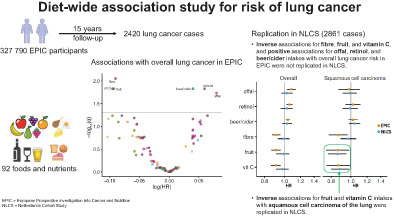

**Abbreviations**
AICR: American Institute for Cancer Research
CI: confidence interval
EPIC: European Prospective Investigation into Cancer and Nutrition
FDR: false discovery rate
GST: glutathione‐S transferase
GWAS: genome‐wide association study
HR: hazard ratio
ICD‐O: International Classification of Diseases for Oncology
ICD‐10: International Classification of Diseases 10th Revision
NLCS: Netherlands Cohort Study
WCRF: World Cancer Research Fund

## 1 INTRODUCTION

Lung cancer (including nonsmall cell and small cell lung cancer) is the most frequently diagnosed cancer in men and third most common cancer in women, and remains the leading cause of cancer death.^1^ Tobacco smoking is overwhelmingly the main risk factor for lung cancer, but other factors such as genetic susceptibility, occupational exposures, air pollution, radon, lifestyle factors and diet might also play a role.^2^

The World Cancer Research Fund/American Institute for Cancer Research (WCRF/AICR) Third Expert Report on diet, nutrition, physical activity and lung cancer suggested that certain dietary factors might contribute to the risk of primary lung cancer.^3^ In particular, the report concluded there is some (albeit limited) evidence that drinking alcohol and consumption of red meat and processed meat might be associated with a higher risk of lung cancer, while consuming foods containing retinol, beta‐carotene or carotenoids might be associated with a lower risk.^3^ Since smoking is the predominant risk factor for lung cancer, diet‐lung cancer associations might be confounded by or differ according to smoking status.^2^, ^4^ It is also possible that dietary factors modify the effects of tobacco exposure, and protective or harmful effects might be restricted to or greater in smokers.^3^, ^5^ There is some evidence of lower lung cancer risk associated with higher intake of fruit and vegetables in current and former smokers,^4^ foods containing vitamin C in current smokers and isoflavones in never smokers.^3^

There remains uncertainty about the role of diet in primary lung cancer risk, with the bulk of existing data stemming from case‐control studies (which are prone to recall and selection biases) or studies that employed suboptimal assessment methods of dietary intake, were underpowered, and/or did not adequately adjust for smoking or examine associations by smoking status or for different histological subtypes of lung cancer.

We sought to evaluate a comprehensive list of individual foods and nutrients in relation to risk of lung cancer using a diet‐wide association study approach.^6^, ^7^, ^8^, ^9^, ^10^, ^11^ Based on the strategy of genome‐wide association studies (GWAS), associations for each dietary factor under investigation are separately estimated, and multiple comparison adjustments are used to select associations to be assessed for replication in an independent study.^12^

## 2 METHODS

### 2.1 Study populations

#### 2.1.1 EPIC

The European Prospective Investigation into Cancer and Nutrition (EPIC) study includes 521 324 men and women, mostly aged 35 to 70 years at recruitment (1992‐2000), from 23 centres in 10 European countries (Denmark, France, Germany, Greece, Italy, the Netherlands, Norway, Spain, Sweden and the United Kingdom). Full details of the study have been described elsewhere.^13^, ^14^ Participants completed questionnaires on diet, lifestyle and medical history at recruitment.

The current analysis did not include data from Greece and excluded participants with a diagnosis of cancer (other than nonmelanoma skin cancer) before recruitment or with missing relevant data.

#### 2.1.2 NLCS

The Netherlands Cohort Study (NLCS) includes 120 852 participants aged 55 to 69 years when recruited in 1986 from the general population of 204 municipalities in the Netherlands.^15^ At recruitment, participants completed a self‐administered questionnaire on dietary habits, lifestyle factors, medical history, family history of cancer and other risk factors for cancer.

A case‐cohort approach was used in the NLCS for efficiency reasons.^15^ A subcohort of 5000 participants was randomly sampled from the cohort immediately after recruitment, for whom vital status information was acquired biennially to estimate person‐time at risk for the full cohort. Incident cancer cases in the entire cohort were identified by record linkage to cancer and pathology registries. This analysis excluded participants with cancer (other than nonmelanoma skin cancer) prior to recruitment as well as those with incomplete or missing dietary or confounder data.

### 2.2 Outcome ascertainment

In EPIC, cancer and mortality data were obtained from population‐based cancer and mortality registries (in Denmark, Italy, the Netherlands, Norway, Spain, Sweden and the United Kingdom) or a combination of methods including cancer and pathology registries, health insurance records and active follow‐up of participants or their next‐of‐kin (in France, Germany and Naples, Italy).^13^ Incident lung cancer cases were determined according to the International Classification of Diseases, 10th Revision (ICD‐10) and the second edition of the International Classification of Diseases for Oncology (ICD‐O‐2), code C34.

In the NLCS, incident lung cancers were identified by record linkage to the Netherlands Cancer Registry and the Dutch National Pathology Registry^15^ and defined according to ICD‐O‐3 code C34.

### 2.3 Dietary assessment

In the EPIC study, usual diet during the preceding 12 months was assessed at enrolment using validated country‐specific or study centre‐specific dietary questionnaires or food records.^13^, ^16^ The questionnaires were self‐administered in most centres and countries, except Ragusa (Italy) and Spain, where interviewers were used. In Malmö (Sweden), a food record was used for cooked meals and a food frequency questionnaire was used for breakfast and foods consumed between the main meals.^17^ Standardised nutrient intakes were calculated using the EPIC Nutrient Database.^18^ The current analysis included 92 dietary factors (63 foods and 29 nutrients; Appendix S1) for which data were available in the centralised EPIC database for at least eight out of the nine countries. The dietary factors were not mutually exclusive, for example, apple/pear, bananas, berries, citrus fruit, grapes and stone fruit were investigated separately as well as total fruit which included all of these types of fruit. Likewise, total alcohol intake was investigated as well as individual alcoholic beverage types: beer/cider, spirits, wine and fortified wine. In addition, the individual food items also contribute to nutrients.

Information on dietary intake in the NLCS was collected at recruitment using a 150‐item semiquantitative food frequency questionnaire that estimated the average frequency and amounts of foods and beverages habitually consumed in the previous 12 months. The food frequency questionnaire has been validated and tested for reproducibility.^19^, ^20^ Nutrient intakes were calculated by multiplying the frequency of intake by the nutrient content of specified portions based on the Dutch food composition table.^21^ Data were available for the same dietary factors as in EPIC, but replication analyses were only performed in the NLCS for foods and nutrients that were identified to be associated with lung cancer risk in EPIC.

### 2.4 Statistical analysis

Cox proportional hazards regression models with age as the time scale were fitted to estimate hazard ratios (HRs) and 95% confidence intervals (CIs) for a 1 SD higher intake of each food or nutrient per day. Age at recruitment was the entry time and age at cancer diagnosis (except nonmelanoma skin cancer), death, emigration or last follow‐up, whichever occurred first, was the exit time. Intakes of foods and nutrients were adjusted for energy intake using the residual method,^22^ and all dietary factors were entered as scaled standardised variables in separate models, without mutual adjustment. Models were stratified by age at recruitment (in 5‐year categories), study centre (EPIC only), sex and smoking status (never, former, current) and adjusted for number of cigarettes smoked per day (in EPIC: fourths, interacted with smoking status; in NLCS: continuous, centred), cigarette smoking years (in EPIC: fourths, interacted with smoking status; in NLCS: continuous, centred), body mass index (BMI; <20, 20 to <23, 23 to <25, 25 to <30, 30 to <35, ≥35 kg/m^2^), physical activity (in EPIC, Cambridge index: inactive, moderately inactive, moderately active, active; in NLCS, nonoccupational physical activity: ≤30, >30 to 60, >60 to 90, >90 min/day), highest level of education (in EPIC: none/primary school, technical/professional school, secondary school, longer education including university; in NLCS: primary school or lower vocational, secondary or medium vocational, higher vocational or university), family history of lung cancer (no, yes; NLCS only, data unavailable in EPIC), history of diabetes (no, yes) and energy intake (kcal/day, continuous). Visual inspection of the smoothed and scaled Schoenfeld residuals revealed no deviations from proportional hazards.

Dietary factors were selected for replication based on the Benjamini‐Hochberg approach with a false discovery rate (FDR) threshold of 0.05.^12^ Foods and nutrients with associations satisfying this FDR (ie, with *q*‐value ˂0.05) within EPIC were then tested in a case‐cohort sample from the NLCS using the Prentice variation on the Cox proportional hazards model with robust SE estimates to account for the case‐cohort design.^23^ Models were adjusted using the same factors as those used in the initial EPIC analysis (as described above).

For the FDR‐significant foods and nutrients identified in the EPIC study, associations with lung cancer were also assessed (in EPIC and in the NLCS) by smoking status at baseline and sex. Analyses were also performed according to different histological subtypes. Using a nested case‐control sample within the EPIC study (786 cases and 1135 controls in whom cotinine was measured in a prior investigation^24^), the associations for these foods and nutrients were also evaluated with additional adjustment for circulating concentrations of cotinine (fourths, based on the distribution among current smokers) using conditional logistic regression models.

All analyses were performed using R version 4.1.0.^25^

## 3 RESULTS

### 3.1 Study characteristics

Of the 465 076 eligible EPIC participants with complete data on length of follow‐up and without a pre‐baseline cancer diagnosis, 5900 participants who did not complete dietary or lifestyle questionnaires, 9064 with extreme values (top or bottom 1%) of the energy intake to energy requirement ratio and 122 322 participants who had missing values for relevant covariates—mainly due to missing data on detailed smoking variables (70 689 participants)—were excluded, leaving 327 790 participants for analysis. In these participants, 2420 incident invasive lung cancers were identified during a median follow‐up of 15 years. The distribution of baseline characteristics is shown in Table 1. Compared to the total cohort of included participants, cases were more likely to be men, older, current smokers, to smoke more cigarettes per day, to have smoked for longer durations and to have lower educational attainment. Mean intakes of the 92 foods and nutrients included in the diet‐wide association study are shown for all participants and by smoking status in Table S1.

**TABLE 1:** Distribution of baseline demographic characteristics and covariates for participants in the European Prospective Investigation into Cancer and Nutrition included in the diet‐wide association study of lung cancer

	Total		Cases	
	N	%	N	%
Total	327 790	100	2420	100
*Sex*				
Women	235 038	72	1241	51
Men	92 752	28	1179	49
*Age at recruitment (years)*				
[17.8, 40)	43 329	13	51	2
[40, 45)	45 889	14	127	5
[45, 50)	55 142	17	190	8
[50, 55)	74 294	23	596	25
[55, 60)	55 478	17	640	26
[60, 65)	42 059	13	704	29
[65, 70)	9428	3	94	4
[70, 75)	1801	0.5	13	0.5
[75, 98.5]	370	0.1	5	0.2
*Smoking status*				
Never	194 087	59	278	11
Former	68 129	21	453	19
Current	65 574	20	1689	70
*Education*				
None/primary school	97 043	30	1130	47
Technical/professional school	77 228	24	690	29
Secondary school	71 099	22	280	12
Longer education, including university degree	82 420	25	320	13
*BMI (kg/m* ^*2*^ *)*				
[10.2, 20)	21 279	6	140	6
[20, 23)	82 374	25	509	21
[23, 25)	68 292	21	497	21
[25, 30)	113 418	35	933	39
[30, 35)	33 452	10	270	11
[35, 77.9]	8975	3	71	3
*Physical activity (Cambridge index)*				
Inactive	60 394	18	535	22
Moderately inactive	112 119	34	786	32
Moderately active	89 745	27	567	23
Active	65 532	20	532	22
*Diabetes*				
No	319 228	97	2337	97
Yes	8562	3	83	3
*Cigarettes per day (fourths)*				
[0, 7.2)	229 940	70	459	19
[7.2, 11.7)	33 897	10	364	15
[11.7, 18.8)	33 880	10	857	35
[18.8, 101]	30 073	9	740	31
*Smoking duration (fourths*, *years)*				
[0, 14)	222 916	68	339	14
[14, 24)	35 791	11	169	7
[24, 33)	34 440	11	431	18
[33, 71.5]	34 643	11	1481	61

In the NLCS, 3860 incident invasive lung cancer cases were identified during up to 20.3 years of follow‐up. Participants with incomplete or inconsistent dietary data (599 cases, 690 subcohort members) and those with missing data on confounders (400 cases and 364 subcohort members) were excluded, leaving 2861 invasive lung cancer cases (including 111 subcohort cases) and 3609 noncase subcohort participants in the current analysis. Participant characteristics are presented in Table 2. Compared to EPIC, the NLCS included a higher proportion of men and participants were on average older and more likely to be smokers.

**TABLE 2:** Distribution of baseline demographic characteristics and covariates for participants in the Netherlands Cohort Study included in the replication analyses (subcohort and lung cancer cases)

	Subcohort noncases		Cases	
	N	%	N	%
Total	3609	100	2861	100
*Sex*				
Women	1736	48	2413	84
Men	1873	52	448	16
*Age at recruitment (years)*				
[55, 60)	1405	39	1051	37
[60, 65)	1250	35	1004	35
[65, 69]	954	26	806	28
*Smoking status*				
Never	1357	38	198	7
Former	1325	37	886	31
Current	927	26	1777	62
*Education*				
Primary school	979	27	866	30
Lower vocational school	783	22	663	23
Secondary, medium vocational school	1312	36	959	34
Higher vocational, University	535	15	373	13
*BMI (kg/m* ^*2*^ *)*				
[14.5, 20)	127	4	99	3
[20, 23)	754	21	614	21
[23, 25)	1102	31	878	31
[25, 30)	1398	39	1150	40
[30, 35)	201	6	115	4
[35, 44.3]	27	0.7	5	0.2
*Physical activity (nonoccupational*, *min/day)*				
[0, 30]	718	20	619	22
(30, 60]	1134	31	872	30
(60, 90]	788	22	544	19
(90, 424]	969	27	826	29
*Diabetes*				
No	3490	97	2770	97
Yes	119	3	91	3
*Family history of lung cancer*				
No	3256	91	2490	87
Yes	353	10	371	13

In smokers	Mean	SD	Mean	SD
Cigarettes per day	15.1	10.1	19.7	10.3
Smoking duration (years)	31.2	12.2	40.0	9.2

### 3.2 Diet‐wide association study in EPIC

**FIGURE 1:**
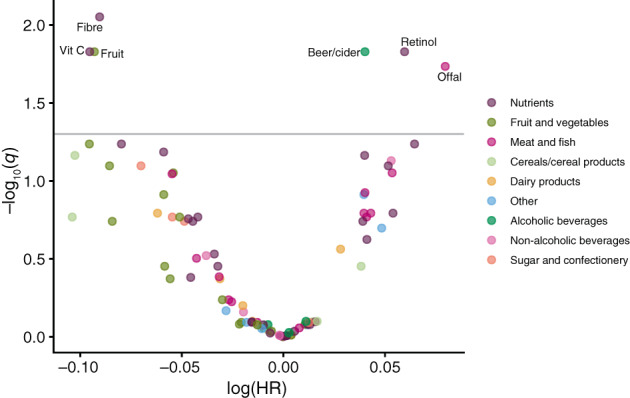
Volcano plot of estimates and *q*‐values from the diet‐wide association study of 92 foods and nutrients in relation to lung cancer risk in the European Prospective Investigation into Cancer and Nutrition. The *y*‐axis is the negative log_10_ transformation of the estimated *q*‐value, and the *x*‐axis is the estimated log hazard ratio for lung cancer in relation to a 1 SD increment in intake per day. The horizontal line indicates the false discovery rate threshold of 0.05. Each dietary factor was analysed one at a time, and ordered left to right according to the lowest to highest HR. Estimates are from Cox proportional hazards models stratified by age at recruitment (<40, 40 to <45, 45 to <50, 50 to <55, 55 to <60, 60 to <65, 65 to <70, 70 to <75, ≥75 years), study centre, sex and smoking status (never, former, current) and adjusted for number of cigarettes smoked per day (fourths) interacted with smoking status, cigarette smoking years (fourths) interacted with smoking status, body mass index (<20, 20 to <23, 23 to <25, 25 to <30, 30 to <35, ≥35 kg/m^2^), physical activity (inactive, moderately inactive, moderately active, active), highest level of education (none/primary school, technical/professional school, secondary school, longer education including university), history of diabetes (no, yes) and energy intake (kcal/day, continuous). The six dietary factors that were carried forward for replication in the NLCS are labelled

Of the 92 foods and nutrients that were evaluated in the EPIC study, six were associated with risk of lung cancer using the FDR threshold of 0.05 (Figure 1 and Appendix S1). There were inverse associations with lung cancer risk for consumption of fibre (HR for a 1 SD increment in intake per day = 0.91, 95% CI 0.87‐0.96), fruit (HR = 0.91, 95% CI 0.86‐0.96) and vitamin C (HR = 0.91, 95% CI 0.86‐0.96). Intakes of offal (HR for a 1 SD increment in intake per day = 1.08, 95% CI 1.03‐1.14), retinol (HR = 1.06, 95% CI 1.03‐1.10) and beer/cider (HR = 1.04, 1.02‐1.07) were positively associated with lung cancer risk.

The analyses in the nested case‐control sample additionally adjusted for circulating cotinine concentrations supported these associations (albeit with wide CIs), with the exception of fibre intake which was not associated with lung cancer risk after adjustment for cotinine (Figure S1).

**FIGURE 2:**
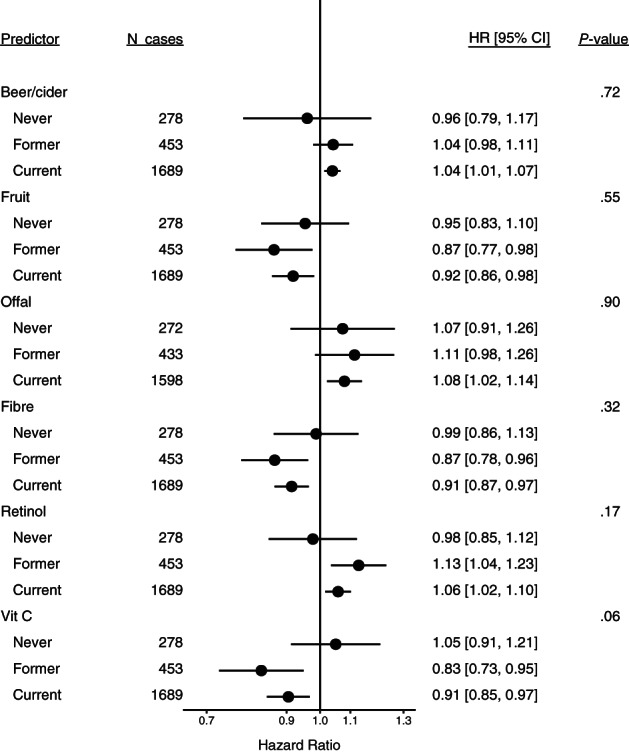
Estimated hazard ratios and 95% confidence intervals for lung cancer in relation to intakes of the six foods and nutrients identified in the European Prospective Investigation into Cancer and Nutrition, by smoking status at baseline. Estimates are for a 1 SD increment in intake per day, from Cox proportional hazards models stratified by age at recruitment (<40, 40 to <45, 45 to <50, 50 to <55, 55 to <60, 60 to <65, 65 to <70, 70 to <75, ≥75 years), study centre and sex, and adjusted for number of cigarettes smoked per day (fourths) interacted with smoking status, cigarette smoking years (fourths) interacted with smoking status, body mass index (<20, 20 to <23, 23 to <25, 25 to <30, 30 to <35, ≥35 kg/m^2^), physical activity (inactive, moderately inactive, moderately active, active), highest level of education (none/primary school, technical/professional school, secondary school, longer education/university), history of diabetes (no, yes) and energy intake (kcal/day, continuous)

**FIGURE 3:**
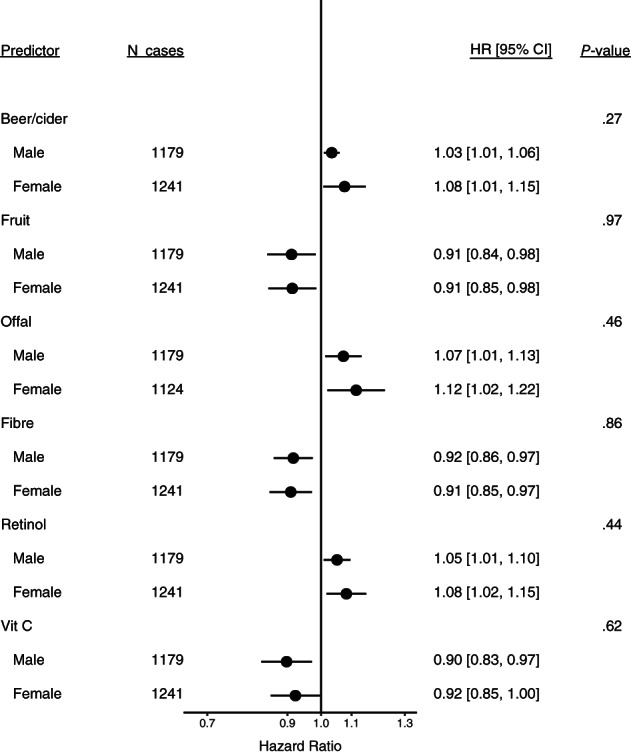
Estimated hazard ratios and 95% confidence intervals for lung cancer in relation to intakes of the six foods and nutrients identified in the European Prospective Investigation into Cancer and Nutrition, by sex. Estimates are for a 1 SD increment in intake per day, from Cox proportional hazards models stratified by age at recruitment (<40, 40 to <45, 45 to <50, 50 to <55, 55 to <60, 60 to <65, 65 to <70, 70 to <75, ≥75 years), study centre, and smoking status (never, former, current) and adjusted for number of cigarettes smoked per day (fourths) interacted with smoking status, cigarette smoking years (fourths) interacted with smoking status, body mass index (<20, 20 to <23, 23 to <25, 25 to <30, 30 to <35, ≥35 kg/m^2^), physical activity (inactive, moderately inactive, moderately active, active), highest level of education (none/primary school, technical/professional school, secondary school, longer education/university), history of diabetes (no, yes) and energy intake (kcal/day, continuous)

The inverse associations for intakes of fibre, fruit and vitamin C, and positive associations for intakes of retinol and beer/cider, were present in former and current smokers but not in never smokers; however, there were only 278 lung cancer cases among never smokers, and *P*‐values for heterogeneity by smoking status were large, except for vitamin C (*P* = .06) (Figure 2). Associations did not differ substantially by sex (*P* ≥ .27) (Figure 3).

**FIGURE 4:**
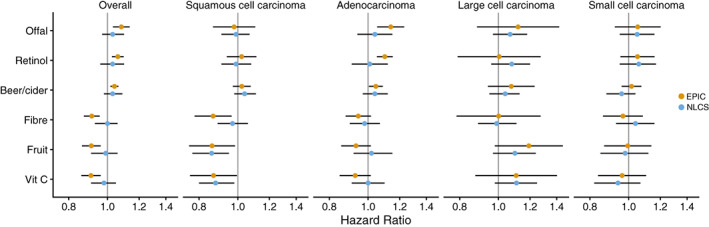
Estimated hazard ratios and 95% confidence intervals for lung cancer in relation to intakes of the six foods and nutrients identified in the European Prospective Investigation into Cancer and Nutrition (yellow), and replication in the Netherlands Cohort Study (blue), overall and for different histological subtypes. Estimates are for a 1 SD increment in intake per day, from Cox proportional hazards models stratified by age at recruitment (in 5‐year categories), study centre (EPIC only), sex and smoking status (never, former, current) and adjusted for number of cigarettes smoked per day (in EPIC: fourths, interacted with smoking status; in NLCS: continuous, centred), cigarette smoking years (in EPIC: fourths, interacted with smoking status; in NLCS: continuous, centred), body mass index (<20, 20 to <23, 23 to <25, 25 to <30, 30 to <35, ≥35 kg/m^2^), physical activity (in EPIC, Cambridge index: inactive, moderately inactive, moderately active, active; in NLCS, nonoccupational physical activity: ≤30, >30 to 60, >60 to 90, >90 min/day), highest level of education (in EPIC: none/primary school, technical/professional school, secondary school, longer education including university; in NLCS: primary school or lower vocational, secondary or medium vocational, higher vocational or university), family history of lung cancer (no, yes; NLCS only), history of diabetes (no, yes) and energy intake (kcal/day, continuous). For offal, only liver consumption was available in the NLCS

In the analyses by histological subtype of lung cancer, intakes of fibre, fruit and vitamin C appeared to be inversely associated with squamous cell carcinoma (HRs per 1 SD increment in intake per day = 0.87, 95% CI 0.78‐0.97; 0.86, 95% CI 0.76‐0.98; and 0.87, 95% CI 0.76‐1.00, respectively), weakly inversely associated with adenocarcinoma (HRs = 0.95, 95% CI 0.88‐1.02; 0.93, 95% CI 0.86‐1.02; 0.93, 95% CI 0.85‐1.02), and not associated with small cell carcinoma (Figure 4 and Table S2). There were no substantial differences in the associations by histological subtype for intakes of the other foods and nutrients identified in the diet‐wide association study (offal, retinol and beer/cider).

Pairwise correlations for the 92 foods and nutrients are displayed in Appendix S2. Fruit intake was correlated with intakes of vitamin C (0.71) and fibre (0.52). There were also notable correlations between intakes of fibre and vitamin C (0.58), and between offal and retinol (0.67).

### 3.3 Replication in the NLCS

In the replication analysis for overall lung cancer, none of the associations for the six dietary factors that were identified in the EPIC study were confirmed in the NLCS (Figure 4 and Tables S3 and S4). The positive association for beer/cider intake was of similar magnitude and in the same direction, while associations for intakes of offal and retinol were weaker in the NLCS, but estimates were accompanied by considerable uncertainty. Despite inverse associations in the EPIC study, intakes of fibre, fruit and vitamin C did not appear to be associated with risk of overall lung cancer in the NLCS. However, in analyses by histological subtype of lung cancer, intakes of fruit and vitamin C were inversely associated with squamous cell carcinoma (HR per 1 SD increment in intake/day = 0.86, 95% CI 0.77‐0.95 and 0.88, 95% CI 0.80‐0.98, respectively) (Figure 4 and Table S2).

## 4 DISCUSSION

In this systematic evaluation of 92 foods and nutrients and risk of lung cancer, higher intakes of fibre, fruit and vitamin C were associated with a 9% lower risk per SD increment in daily intake, while offal, retinol and beer/cider intakes were associated with a higher risk of lung cancer in the EPIC study. The inverse associations for fibre, fruit and vitamin C intakes, and positive association for retinol intake with lung cancer risk were evident in former and current smokers but not in never smokers. The associations with overall lung cancer were not replicated when assessed in the independent NLCS. However, when considering histological subtypes of lung cancer, inverse associations observed in EPIC were replicated in the NLCS for intakes of fruit and vitamin C with squamous cell carcinoma.

In a nested case‐control sample of EPIC participants, associations for the six identified dietary factors were in the same direction and of similar magnitude after adjusting for circulating cotinine concentrations, apart from fibre intake, for which there was no association. Nevertheless, the association for fibre was null in the NLCS, and this is consistent with the WCRF/AICR report which found a lack of evidence to suggest that dietary fibre intake is associated with primary lung cancer risk.^3^

In EPIC, there was a higher risk of lung cancer associated with beer/cider consumption, but not with other alcoholic beverage types (wine, spirits) or total alcohol intake. Although a meta‐analysis found a positive association between beer and lung cancer risk in those consuming an average of one or more drinks per day,^26^ evidence from previous studies has suggested a higher risk of lung cancer for overall alcohol consumption but not beer,^3^ however most studies have not distinguished between types of alcoholic drinks. Because smoking and alcohol consumption are strongly correlated, the relationship between alcohol and lung cancer risk reported in many studies, even after adjustment for smoking, might be biased by residual confounding. A meta‐analysis found no association between alcohol consumption and lung cancer risk in never smokers.^27^ In the current analysis, beer/cider intake was positively associated with lung cancer risk in current smokers (with an identical point estimate for former smokers), but not associated with lung cancer in never smokers.

Associations with intakes of other dietary factors in EPIC also appeared to differ according to smoking status, with inverse associations for fibre, fruit and vitamin C, and a positive association for retinol in former and current smokers but not in never smokers. The findings for fruit and vitamin C are consistent with the WCRF/AICR report, which identified some evidence of lower lung cancer risk for higher consumption of fruit in current and former smokers, and vitamin C in current smokers.^3^ A meta‐analysis similarly found an inverse association between fruit intake and lung cancer risk in smokers but not in never smokers.^4^ Fruit is a source of vitamin C as well as other antioxidants and various phytochemicals which might ameliorate some of the effects of tobacco exposure in multiple pathways involved in lung carcinogenesis.^3^ Antioxidant‐rich foods might have greater benefits in smokers, and this might explain why the observed associations were restricted to ever smokers and squamous cell carcinoma.^5^ The higher risk of lung cancer associated with higher intake of retinol in EPIC is inconsistent with previous studies suggesting no association for dietary retinol intake and an inverse association for serum retinol concentrations.^3^ However, long‐term use of retinol supplements was associated with a higher risk of lung cancer in the VITamins And Lifestyle (VITAL) cohort study,^28^ and there is evidence that taking high‐dose beta‐carotene supplements (a retinol precursor) is associated with a higher risk of lung cancer in current and former smokers.^3^, ^29^, ^30^ Our findings of a positive association between retinol intake and lung cancer risk in ever smokers but not in never smokers suggests that retinol potentially modifies the effects of tobacco exposure. It is thought that there is an interaction between smoking, beta‐carotene and glutathione‐S transferase (GST) genetic variants such that high‐dose beta‐carotene supplementation—potentially leading to supra‐physiological concentrations of beta‐carotene—confers a higher risk of lung cancer mainly in heavy smokers without the GSTM1 variant, thereby having reduced ability to metabolise certain toxins and carcinogens including those derived from tobacco smoke.^3^ Taken together, our findings suggest that diet modifies the effect of tobacco exposure and dietary factors are likely to be more relevant for lung cancer risk in smokers than in never smokers.

Although the association between beer/cider consumption and lung cancer risk in EPIC did not notably differ by histological subtype, a higher risk of adenocarcinoma was apparent while there was little evidence of an association for squamous cell carcinoma or small cell carcinoma. By contrast, in a pooled analysis of data from 21 case‐control studies and one cohort study (a previous EPIC analysis) from the International Lung Cancer Consortium and the SYNERGY Consortium, there was no increased risk of lung cancer associated with high total alcohol consumption, but when considering histological subtypes and beverage types, beer intake was positively associated with risk of squamous cell carcinoma of the lung (OR for ≥20 g/day vs nondrinkers = 1.42, 95% CI 1.06‐1.90).^31^ In our study, protective effects of fruit and vitamin C (and fibre in EPIC) were observed for squamous cell carcinoma, which among nonsmall cell lung cancers is the subtype most strongly associated with smoking.^32^ Meta‐analyses have found associations in the direction of a lower risk of squamous cell carcinoma, adenocarcinoma and small cell carcinoma of the lung for higher fruit intake but estimates were based on few studies and accompanied by substantial uncertainty.^4^, ^33^ Further investigations in large prospective studies or consortia are required to determine whether putative dietary factors are associated with specific histological subtypes of lung cancer.

The positive association between offal intake and lung cancer risk identified in EPIC was not replicated in the NLCS. A meta‐analysis of four studies found no association between offal intake and lung cancer risk in nonsmokers,^34^ but higher offal consumption was associated with a higher risk of lung cancer among heavy smokers participating in a lung cancer screening programme in Italy.^35^ Previous studies have found a higher risk of lung cancer associated with higher red meat and processed meat consumption,^3^, ^36^, ^37^, ^38^ including among nonsmokers (for red meat),^34^ whereas no association was found in the current study. Although there are plausible mechanisms linking meat consumption with carcinogenesis (albeit also often mediated by cooking methods used),^36^ mechanisms specific to lung cancer have not been identified.^3^ The lack of association using the systematic diet‐wide association study approach suggests that red or processed meat consumption is unlikely to have a major effect on lung cancer risk.

Strengths of our study include the large study population with many lung cancer cases, long follow‐up duration, wide variation in diet, extensive information on potential confounders and the systematic approach which assessed a comprehensive set of foods and nutrients while accounting for multiple comparisons, and replication of findings in an independent cohort. Further strengths were the ability to examine associations with further adjustment for circulating concentrations of cotinine and according to smoking status, sex and histological subtype. The main limitation was that analyses were based on a single assessment of diet at recruitment and changes in dietary habits were not considered. For consistency, analyses of each food and nutrient were adjusted for total energy intake, but this introduces a nonspecified substitution model for energy‐providing foods and nutrients, which is not biologically optimal. The interpretation of results is therefore not straightforward; for example, for smoking antagonists, absolute intake may be more relevant. Because of the nature of the diet‐wide association study approach, dietary patterns were not evaluated. In addition, mutual adjustment for dietary exposures was not performed and intercorrelations were not accounted for. Since many of the dietary items share common sources of intake, correlations of approximately 0.5 to 0.7 were found between several of the foods and nutrients associated with lung cancer risk in EPIC, and it is therefore difficult to disentangle their independent effects. Although analyses were rigorously adjusted for smoking habits, residual confounding by smoking cannot be ruled out. A strength of the EPIC study is the large variability in dietary intakes of individual foods and nutrients,^13^, ^14^ whereas on average there are narrower distributions of intake in the NLCS, and thus there may have been greater chances of observing associations in the EPIC study than in the replication analyses in the NLCS. Although the NLCS included a greater proportion of men and smokers, analyses by sex and smoking status suggested these differences in study characteristics are unlikely to explain the lack of replication of EPIC results in the NLCS. Finally, while the diet‐wide association study approach has merits, intakes of the foods and nutrients are not independent and were included based on their availability in the EPIC database. It is possible that certain dietary factors other than the 92 foods and nutrients included in this investigation might be associated with lung cancer risk.

In summary, although associations with lung cancer risk were found for six dietary factors, namely fibre, fruit, vitamin C, offal, retinol and beer/cider in the EPIC study, these were not supported in the NLCS except for inverse associations of fruit and vitamin C intakes with squamous cell carcinoma of the lung.

## AUTHOR CONTRIBUTIONS

**Konstantinos K. Tsilidis**: Funding acquisition and supervision; **David C. Muller, Konstantinos K. Tsilidis and Ioanna Tzoulaki**: Conceptualisation and methodology; **Piet A. van den Brandt**: Data curation and formal analysis in the NLCS; **David C. Muller**: Formal analysis in EPIC and visualisation; **Alicia K. Heath**: Writing ‐ original draft. All authors contributed to writing ‐ review & editing. The work reported in the article has been performed by the authors, unless clearly specified in the text.

## FUNDING INFORMATION

This work was supported by the World Cancer Research Fund International Regular Grant Programme (WCRF 2014/1180 to Konstantinos K. Tsilidis).

The coordination of EPIC is financially supported by International Agency for Research on Cancer (IARC) and also by the Department of Epidemiology and Biostatistics, School of Public Health, Imperial College London which has additional infrastructure support provided by the NIHR Imperial Biomedical Research Centre (BRC).

The national cohorts are supported by: Danish Cancer Society (Denmark); Ligue Contre le Cancer, Institut Gustave Roussy, Mutuelle Générale de l'Education Nationale, Institut National de la Santé et de la Recherche Médicale (INSERM) (France); German Cancer Aid, German Cancer Research Center (DKFZ), German Institute of Human Nutrition Potsdam‐Rehbruecke (DIfE), Federal Ministry of Education and Research (BMBF) (Germany); Associazione Italiana per la Ricerca sul Cancro‐AIRC‐Italy, Compagnia di SanPaolo and National Research Council (Italy); Dutch Ministry of Public Health, Welfare and Sports (VWS), Netherlands Cancer Registry (NKR), LK Research Funds, Dutch Prevention Funds, Dutch ZON (Zorg Onderzoek Nederland), World Cancer Research Fund (WCRF), Statistics Netherlands (The Netherlands); Health Research Fund (FIS) ‐ Instituto de Salud Carlos III (ISCIII), Regional Governments of Andalucía, Asturias, Basque Country, Murcia and Navarra and the Catalan Institute of Oncology ‐ ICO (Spain); Swedish Cancer Society, Swedish Research Council and County Councils of Skåne and Västerbotten (Sweden); Cancer Research UK (14136 to EPIC‐Norfolk; C8221/A29017 to EPIC‐Oxford), Medical Research Council (1000143 to EPIC‐Norfolk; MR/M012190/1 to EPIC‐Oxford) (United Kingdom).

## CONFLICT OF INTEREST

The authors declare no potential conflicts of interest.

## ETHICS STATEMENT

Written informed consent was provided by all participants. Ethical approval for the EPIC study was obtained from the ethical review board of the International Agency for Research on Cancer and from local ethics committees in each participating country. The NLCS was approved by the institutional review boards of the Nederlandse Organisatie voor Toegepast Natuurwetenschappehlijk Onderzoek (TNO) Quality of Life research institute (Zeist, Netherlands) and Maastricht University (Maastricht, Netherlands).

## Supporting information


**Appendix S1** Supporting Information.

**Appendix S2** Supporting Information.

**Appendix S3** Supporting Information Tables and Figures.

## Data Availability

For information on how to submit an application for gaining access to EPIC data and/or biospecimens, please follow the instructions at http://epic.iarc.fr/access/index.php. Further details and other data that support the findings of our study are available from the corresponding author upon request.
