# Supplementary material for: Diet‐wide association study of 92 foods and nutrients and lung cancer risk in the European Prospective Investigation into Cancer and Nutrition study and the Netherlands Cohort Study
Source: Int J Cancer. 2022 Aug 4;151(11):1935–46. doi: 10.1002/ijc.34211 (PMC9804326; doi:10.1002/ijc.34211)

Diet-wide association study of 92 foods and nutrients and lung cancer risk in the European Prospective Investigation into Cancer and Nutrition study and the Netherlands Cohort Study

Alicia K. Heath, David C. Muller, Piet A. van den Brandt, Elena Critselis, Marc Gunter, Paolo Vineis, Elisabete Weiderpass, Heiner Boeing, Pietro Ferrari, Melissa A. Merritt, Agnetha L. Rostgaard-Hansen, Anne Tjønneland, Kim Overvad, Verena Katzke, Bernard Srour, Giovanna Masala, Carlotta Sacerdote, Fulvio Ricceri, Fabrizio Pasanisi, Bas Bueno-de-Mesquita, George S. Downward, Guri Skeie, Torkjel M. Sandanger, Marta Crous-Bou, Miguel Rodríguez-Barranco, Pilar Amiano, José María Huerta, Eva Ardanaz, Isabel Drake, Mikael Johansson, Ingegerd Johansson, Tim Key, Nikos Papadimitriou, Elio Riboli, Ioanna Tzoulaki, Konstantinos K. Tsilidis

Supplementary Figures and Tables

Supplementary Table 1: Mean (standard deviation) intakes of the 92 foods and nutrients investigated in relation to risk of lung cancer in the European Prospective Investigation into Cancer and Nutrition. 2

Supplementary Table 2: Hazard ratios and 95% confidence intervals for lung cancer in EPIC and the NLCS in relation to intakes of the six foods and nutrients identified in the diet-wide association study, overall and for different histological subtypes. 6

Supplementary Table 3: Hazard ratios and 95% confidence intervals for lung cancer in the NLCS in relation to intakes of the six foods and nutrients identified in the diet-wide association study in EPIC, by smoking status at baseline. 7

Supplementary Table 4: Hazard ratios and 95% confidence intervals for lung cancer in the NLCS in relation to intakes of the six foods and nutrients identified in the diet-wide association study in EPIC, by sex. 8

Supplementary Figure 1: Estimated rate ratios and 95% confidence intervals of lung cancer for intakes of the six foods and nutrients identified in the European Prospective Investigation into Cancer and Nutrition, before and after adjusting for circulating concentrations of cotinine. 9

Separate files:

Appendix 1: Estimates from Cox regression models for a one standard deviation increment in intake of 92 foods and nutrients in relation to risk of lung cancer in the European Prospective Investigation into Cancer and Nutrition.

Appendix 2: Pairwise correlations for the 92 foods and nutrients included in the diet-wide association study of lung cancer risk in the European Prospective Investigation into Cancer and Nutrition.

Supplementary Table 1: Mean (standard deviation) intakes of the 92 foods and nutrients investigated in relation to risk of lung cancer in the European Prospective Investigation into Cancer and Nutrition.

Food/nutrient	Overall, N = 327,790	Smoking status<br>Never, N = 194,087	Former, N = 68,129	Current, N = 65,574
Apple/pear (g/d)	72 (87)	71 (88)	84 (92)	64 (81)
Banana (g/d)	24 (37)	25 (38)	27 (38)	20 (32)
Beef (g/d)	16 (19)	14 (17)	19 (21)	20 (21)
Beer/cider (g/d)	94 (246)	59 (158)	130 (289)	158 (365)
Berries (g/d)	8 (12)	9 (13)	7 (11)	6 (10)
Breakfast cereal (g/d)	16 (41)	19 (45)	15 (39)	8 (24)
Biscuits (g/d)	7 (13)	7 (13)	7 (13)	6 (12)
Bread (g/d)	127 (79)	121 (78)	136 (80)	137 (81)
Non-white bread (g/d)	69 (74)	58 (71)	89 (76)	80 (74)
White bread (g/d)	53 (74)	57 (75)	42 (68)	52 (75)
Butter (g/d)	4 (8)	4 (8)	4 (8)	4 (8)
Cabbage (g/d)	23 (31)	23 (31)	25 (34)	19 (27)
Cake (g/d)	34 (39)	37 (40)	31 (38)	28 (37)
Cheese (g/d)	38 (34)	39 (34)	38 (33)	37 (33)
Chocolate (g/d)	8 (14)	8 (14)	8 (13)	8 (14)
Citrus fruit (g/d)	50 (64)	52 (64)	50 (65)	44 (64)
Coffee (g/d)	381 (373)	316 (314)	414 (377)	538 (467)
Cream puddings (g/d)	14 (23)	15 (24)	13 (21)	12 (20)
Crispbread (g/d)	8 (16)	9 (18)	6 (13)	5 (12)
Eggs (g/d)	18 (17)	18 (17)	18 (16)	20 (18)
Fish (g/d)	29 (32)	28 (29)	30 (34)	33 (36)
Fatty fish (g/d)	12 (15)	11 (14)	12 (16)	13 (16)
Lean fish (g/d)	18 (24)	17 (22)	19 (26)	21 (28)
Fish products (g/d)	6 (9)	5 (8)	6 (9)	6 (9)
Fortified wine (g/d)	4 (15)	4 (12)	4 (18)	4 (21)
Fruit (g/d)	231 (181)	247 (182)	227 (180)	186 (170)
Fruiting vegetables (g/d)	61 (52)	63 (53)	60 (51)	54 (50)

Food/nutrient	Overall, N = 327,790	Smoking status<br>Never, N = 194,087	Former, N = 68,129	Current, N = 65,574
Fruit/vegetable juice (g/d)	68 (117)	68 (113)	72 (123)	64 (122)
Grapes (g/d)	10 (15)	11 (16)	10 (15)	8 (13)
Ice cream (g/d)	7 (11)	7 (11)	8 (12)	6 (11)
Lamb (g/d)	3.6 (7.3)	4.2 (7.9)	2.7 (5.9)	2.9 (6.6)
Leafy vegetables (g/d)	30 (40)	34 (42)	24 (35)	25 (38)
Legumes (g/d)	16 (25)	16 (24)	14 (24)	15 (28)
Liver (g/d)	2.2 (4.4)	2.2 (4.7)	1.9 (3.4)	2.4 (4.2)
Margarine (g/d)	11 (15)	11 (15)	11 (13)	12 (14)
Vegetable margarine (g/d)	7 (12)	7 (12)	6 (11)	6 (10)
Mayonnaise (g/d)	2.7 (5.8)	2.5 (5.6)	3.0 (6.2)	3.0 (5.9)
Molluscs (g/d)	3.5 (5.9)	3.4 (5.9)	3.6 (5.9)	3.9 (6.0)
Mushrooms (g/d)	5.6 (8.6)	6.0 (8.8)	5.4 (8.6)	4.6 (7.6)
Nuts (g/d)	3.9 (8.5)	3.8 (8.1)	4.2 (9.3)	3.7 (8.8)
Offal (g/d)	2.8 (5.8)	2.9 (6.5)	2.2 (4.0)	2.9 (5.0)
Onion/garlic (g/d)	12 (13)	10 (13)	13 (14)	14 (14)
Pasta/grains (g/d)	66 (67)	66 (62)	69 (75)	65 (72)
Pod vegetables (g/d)	9 (12)	10 (13)	8 (12)	6 (9)
Pork (g/d)	14 (17)	12 (15)	16 (18)	19 (20)
Potato (g/d)	90 (72)	87 (71)	91 (69)	98 (75)
Poultry (g/d)	19 (20)	19 (20)	20 (20)	20 (20)
Processed meat (g/d)	34 (32)	31 (29)	35 (35)	39 (35)
Other processed meat (g/d)	15 (20)	14 (18)	17 (21)	19 (23)
Alcohol (g/d)	12 (17)	9 (13)	16 (19)	18 (23)
Calcium (mg/d)	989 (414)	1,002 (412)	990 (408)	951 (427)
Beta carotene (μg/d)	3,423 (2,772)	3,629 (2,767)	3,351 (2,868)	2,886 (2,601)
Carbohydrates (g/d)	230 (75)	231 (73)	231 (75)	227 (79)
Cholesterol (mg/d)	323 (151)	315 (148)	320 (149)	349 (159)
Monounsaturated fats (g/d)	29 (13)	29 (12)	30 (13)	31 (14)
Polyunsaturated fats (g/d)	13.2 (5.9)	13.2 (5.8)	13.2 (5.9)	13.2 (6.1)
Saturated fats (g/d)	31 (13)	31 (13)	30 (12)	31 (13)

Food/nutrient	Overall, N = 327,790	Smoking status<br>Never, N = 194,087	Former, N = 68,129	Current, N = 65,574
Total fats (g/d)	80 (29)	79 (28)	79 (29)	82 (30)
Animal fats (g/d)	37 (18)	36 (18)	37 (18)	39 (19)
Plant fats (g/d)	24 (16)	24 (15)	23 (17)	23 (17)
Iron (mg/d)	13.0 (4.2)	12.9 (4.0)	13.3 (4.2)	13.1 (4.5)
Fibre (g/d)	23 (8)	23 (8)	24 (8)	22 (8)
Potassium (mg/d)	3,626 (1,023)	3,581 (1,008)	3,696 (1,027)	3,683 (1,055)
Magnesium (mg/d)	358 (109)	357 (112)	363 (102)	357 (106)
Phosphorus (mg/d)	1,491 (459)	1,462 (449)	1,539 (460)	1,526 (479)
Total proteins (g/d)	87 (28)	86 (27)	88 (28)	90 (29)
Animal proteins (g/d)	52 (23)	51 (23)	52 (23)	56 (24)
Plant proteins (g/d)	27 (10)	27 (10)	28 (11)	27 (11)
Retinol (µg/d)	816 (714)	798 (703)	796 (672)	891 (782)
Riboflavin (mg/d)	1.82 (0.73)	1.87 (0.75)	1.76 (0.69)	1.74 (0.70)
Starch (g/d)	123 (50)	124 (49)	124 (51)	122 (52)
Sugar (g/d)	103 (43)	104 (42)	102 (43)	99 (48)
Thiamine (mg/d)	1.30 (0.49)	1.32 (0.49)	1.30 (0.50)	1.25 (0.47)
Vitamin B12 (µg/d)	6.6 (3.9)	6.5 (4.1)	6.5 (3.5)	7.0 (3.8)
Vitamin B6 (mg/d)	1.82 (0.59)	1.82 (0.58)	1.84 (0.60)	1.77 (0.60)
Vitamin C (mg/d)	123 (64)	128 (64)	120 (63)	109 (62)
Vitamin D (µg/d)	4.10 (3.39)	3.92 (3.04)	4.28 (3.77)	4.44 (3.89)
Vitamin E (mg/d)	11.7 (5.3)	11.8 (5.3)	11.8 (5.2)	11.4 (5.3)
Red meat (g/d)	43 (36)	39 (34)	45 (37)	52 (39)
Root vegetables (g/d)	25 (30)	26 (30)	25 (31)	20 (28)
Sauces (g/d)	18 (18)	20 (18)	17 (18)	16 (17)
Savoury biscuits (g/d)	3.2 (6.5)	3.1 (6.1)	3.4 (7.0)	3.1 (6.8)
Soft drinks (g/d)	74 (169)	65 (154)	79 (173)	97 (200)
Soups (g/d)	58 (79)	63 (85)	49 (65)	50 (66)
Spirits (g/d)	3.4 (11.8)	2.1 (7.1)	4.7 (13.0)	6.5 (19.3)
Stalk vegetables (g/d)	10 (12)	10 (13)	10 (13)	8 (10)
Stone fruit (g/d)	31 (45)	36 (49)	26 (39)	24 (38)

Food/nutrient	Overall, N = 327,790	Smoking status<br>Never, N = 194,087	 Former, N = 68,129	 Current, N = 65,574
Sugars (g/d)	18 (20)	18 (20)	16 (18)	17 (21)
Tea (g/d)	189 (307)	193 (306)	216 (326)	145 (288)
Total milk (g/d)	186 (206)	185 (199)	182 (205)	194 (226)
Wine (g/d)	77 (133)	62 (109)	98 (149)	98 (168)
Yoghurt (g/d)	63 (86)	72 (90)	56 (80)	46 (80)

Supplementary Table 2: Hazard ratios and 95% confidence intervals for lung cancer in EPIC and the NLCS in relation to intakes of the six foods and nutrients identified in the diet-wide association study, overall and for different histological subtypes.

	Overall	Lung cancer subtype			
		Squamous cell carcinoma	Adenocarcinoma	Large cell carcinoma	Small cell carcinoma
**EPIC**					
Cases[a]	1,921	436	922	83	374
	HR (95% CI)[b]	HR (95% CI)[b]	HR (95% CI)[b]	HR (95% CI)[b]	HR (95% CI)[b]
Offal	1.08 (1.03-1.14)	0.98 (0.87-1.11)	1.14 (1.05-1.23)	1.12 (0.88-1.42)	1.05 (0.92-1.20)
Retinol	1.06 (1.03-1.10)	1.02 (0.94-1.11)	1.10 (1.05-1.15)	1.00 (0.79-1.28)	1.05 (0.95-1.16)
Beer/cider	1.04 (1.02-1.07)	1.02 (0.97-1.08)	1.05 (1.00-1.09)	1.08 (0.94-1.23)	1.02 (0.96-1.08)
Fibre	0.91 (0.87-0.96)	0.87 (0.78-0.97)	0.95 (0.88-1.02)	1.00 (0.79-1.28)	0.97 (0.86-1.08)
Vitamin C	0.91 (0.86-0.96)	0.87 (0.76-1.00)	0.93 (0.85-1.02)	1.11 (0.88-1.40)	0.96 (0.84-1.11)
Fruit	0.91 (0.86-0.96)	0.86 (0.76-0.98)	0.93 (0.86-1.02)	1.19 (0.98-1.45)	0.99 (0.87-1.14)
**NLCS**					
Cases	2,861	1,037	685	445	478
	HR (95% CI)[b]	HR (95% CI)[b]	HR (95% CI)[b]	HR (95% CI)[b]	HR (95% CI)[b]
Offal[c]	1.03 (0.97-1.10)	0.99 (0.91-1.07)	1.04 (0.94-1.15)	1.07 (0.97-1.18)	1.05 (0.95-1.16)
Retinol	1.03 (0.96-1.10)	0.99 (0.91-1.08)	1.01 (0.91-1.12)	1.08 (0.96-1.20)	1.06 (0.95-1.17)
Beer/cider	1.03 (0.98-1.09)	1.04 (0.98-1.11)	1.04 (0.97-1.12)	1.04 (0.95-1.13)	0.96 (0.88-1.04)
Fibre	1.00 (0.93-1.06)	0.97 (0.89-1.06)	0.98 (0.90-1.07)	0.99 (0.89-1.11)	1.04 (0.93-1.16)
Vitamin C	0.98 (0.91-1.05)	0.88 (0.80-0.98)	1.00 (0.91-1.10)	1.11 (0.98-1.25)	0.94 (0.82-1.07)
Fruit	0.99 (0.91-1.06)	0.86 (0.77-0.95)	1.02 (0.92-1.15)	1.10 (0.97-1.24)	0.98 (0.85-1.12)

EPIC, European Prospective Investigation into Cancer and Nutrition; NLCS, Netherlands Cohort Study

[a]106 cases were 'other' subtype.

[b]Estimates are for a one standard deviation increment in intake per day, from Cox proportional hazards models stratified by age at recruitment (in 5-year categories), study centre (EPIC only), sex, and smoking status (never, former, current), and adjusted for number of cigarettes smoked per day (in EPIC: fourths, interacted with smoking status; in NLCS: continuous, centered), cigarette smoking years (in EPIC: fourths, interacted with smoking status; in NLCS: continuous, centered), body mass index (<20, 20-<23, 23-<25, 25-<30, 30-<35, ≥35 kg/m^2), physical activity (in EPIC, Cambridge index: inactive, moderately inactive, moderately active, active; in NLCS, non-occupational physical activity: ≤30, >30-60, >60-90, >90 min/day), highest level of education (in EPIC: none/primary school, technical/professional school, secondary school, longer education including university; in NLCS: primary school or lower vocational, secondary or medium vocational, higher vocational or university), family history of lung cancer (no, yes; NLCS only), history of diabetes (no, yes), and energy intake (kcal/day, continuous).

[c]For offal, only liver consumption was available in the NLCS.

Supplementary Table 3: Hazard ratios and 95% confidence intervals for lung cancer in the NLCS in relation to intakes of the six foods and nutrients identified in the diet-wide association study in EPIC, by smoking status at baseline.

	NLCS		
	**Never smokers**	**Former smokers**	**Current smokers**
Cases	198	886	1,777
	HR (95% CI) per 1 SD[a]	HR (95% CI) per 1 SD[a]	HR (95% CI) per 1 SD[a]
Offal[b]	0.91 (0.75-1.11)	1.14 (1.01-1.28)	1.01 (0.93-1.09)
Retinol	0.93 (0.78-1.10)	1.02 (0.90-1.14)	1.04 (0.95-1.14)
Beer/cider	1.12 (0.81-1.55)	0.99 (0.90-1.10)	1.02 (0.95-1.09)
Fibre	0.97 (0.84-1.12)	0.98 (0.89-1.09)	1.01 (0.92-1.11)
Vitamin C	1.05 (0.89-1.23)	1.02 (0.92-1.14)	0.95 (0.86-1.06)
Fruit	0.92 (0.78-1.09)	1.07 (0.96-1.18)	0.95 (0.85-1.07)

EPIC, European Prospective Investigation into Cancer and Nutrition; NLCS, Netherlands Cohort Study

[a]Estimates are for a one standard deviation increment in intake per day, from Cox proportional hazards models stratified by age at recruitment (50-59, 60-64, 65-69 years), and sex, and adjusted for number of cigarettes smoked per day (continuous, centered), cigarette smoking years (continuous, centered), body mass index (<20, 20-<23, 23-<25, 25-<30, 30-<35, ≥35 kg/m^2), non-occupational physical activity (≤30, >30-60, >60-90, >90 min/day), highest level of education (primary school or lower vocational, secondary or medium vocational, higher vocational or university), family history of lung cancer (no, yes), history of diabetes (no, yes), and energy intake (kcal/day, continuous).

[b]For offal, only liver consumption was available in the NLCS.

Supplementary Table 4: Hazard ratios and 95% confidence intervals for lung cancer in the NLCS in relation to intakes of the six foods and nutrients identified in the diet-wide association study in EPIC, by sex.

	NLCS		
	**Overall**	**Men**	**Women**
Cases	2,861	2,413	448
	HR (95% CI) per 1 SD[a]	HR (95% CI) per 1 SD[a]	HR (95% CI) per 1 SD[a]
Offal[b]	1.03 (0.97-1.10)	1.04 (0.97-1.12)	0.97 (0.84-1.12)
Retinol	1.03 (0.96-1.10)	1.01 (0.93-1.09)	1.12 (0.99-1.27)
Beer/cider	1.03 (0.98-1.09)	1.03 (0.98-1.09)	1.28 (0.92-1.77)
Fibre	1.00 (0.93-1.06)	1.00 (0.93-1.08)	0.99 (0.85-1.16)
Vitamin C	0.98 (0.91-1.05)	0.95 (0.87-1.03)	1.11 (0.96-1.28)
Fruit	0.99 (0.91-1.06)	0.98 (0.90-1.07)	1.03 (0.88-1.20)

EPIC, European Prospective Investigation into Cancer and Nutrition; NLCS, Netherlands Cohort Study

[a]Estimates are for a one standard deviation increment in intake per day, from Cox proportional hazards models stratified by age at recruitment (50-59, 60-64, 65-69 years), and smoking status (never, former, current), and adjusted for number of cigarettes smoked per day (continuous, centered), cigarette smoking years (continuous, centered), body mass index (<20, 20-<23, 23-<25, 25-<30, 30-<35, ≥35 kg/m^2), non-occupational physical activity (≤30, >30-60, >60-90, >90 min/day), highest level of education (primary school or lower vocational, secondary or medium vocational, higher vocational or university), family history of lung cancer (no, yes), history of diabetes (no, yes), and energy intake (kcal/day, continuous).

[b]For offal, only liver consumption was available in the NLCS.

Supplementary Figure 1: Estimated rate ratios and 95% confidence intervals of lung cancer for intakes of the six foods and nutrients identified in the European Prospective Investigation into Cancer and Nutrition, before and after adjusting for circulating concentrations of cotinine. Green points are estimates from the full cohort, unadjusted for cotinine. Yellow points are from conditional logistic regression models using a nested case-control sample, additionally adjusted for four categories of circulating cotinine. Estimates are for a one standard deviation increment in daily intake of each food or nutrient from models stratified by age at recruitment (<40, 40-44, 45-49, 50-54, 55-59, 60-64, 65-69, 70-74, ≥75 years), study centre, sex, and smoking status (never, former, current), and adjusted for number of cigarettes smoked per day (fourths) interacted with smoking status, cigarette smoking years (fourths) interacted with smoking status, body mass index (<20, 20-<23, 23-<25, 25-<30, 30-<35, ≥35 kg/m^2), physical activity (inactive, moderately inactive, moderately active, active), highest level of education (none/primary school, technical/professional school, secondary school, longer education including university), history of diabetes (no, yes), and energy intake (kcal/day, continuous).

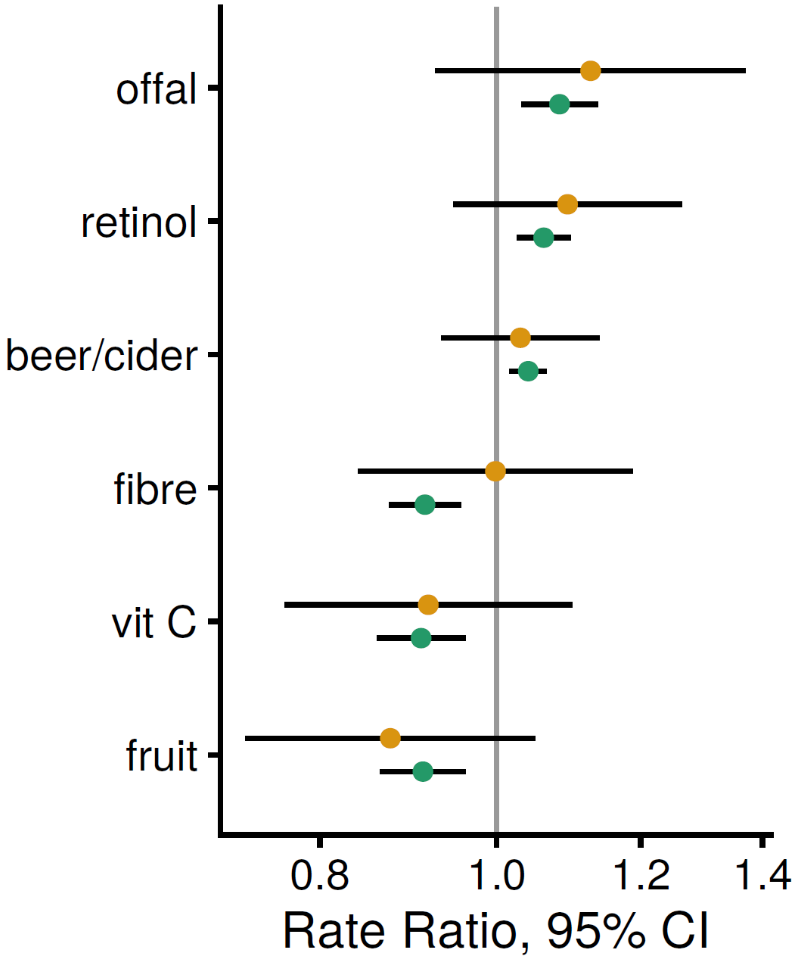

Supplement: Supplementary file 3 — Appendix S3 Supporting Information Tables and Figures. [file IJC-151-1935-s001.pdf]
